# Optimization of Nozzle Inclination and Process Parameters in Air-Shielding Electrochemical Micromachining

**DOI:** 10.3390/mi10120846

**Published:** 2019-12-04

**Authors:** Minghuan Wang, Yongchao Shang, Kailei He, Xuefeng Xu, Guoda Chen

**Affiliations:** Key Laboratory of Special Purpose Equipment and Advanced Processing Technology, Ministry of Education and Zhejiang Province, Zhejiang University of Technology, Hangzhou 310014, China; Shangyc@zjut.edu.cn (Y.S.); 2111802026@zjut.edu.cn (K.H.); xuxuefeng@zjut.edu.cn (X.X.); gchen@zjut.edu.cn (G.C.)

**Keywords:** air-shielding electrochemical micro-machining, nozzle design, flow field, grey relational analysis, optimization

## Abstract

Microstructures on metal surfaces with diameters of tens to hundreds of micrometers and depths of several micrometers to tens of micrometers can improve the performance of engineering parts. Air-shielding electrochemical micromachining (AS-EMM) is a promising method for fabricating these microstructures, owing to its advantage of high efficient and better localization. However, the machining performance is often influenced by the machining or nonmachining parameters in AS-EMM. In order to get a better machining result in AS-EMM, the optimization of AS-EMM, including nozzle inclination and process parameters, was studied in this paper. Firstly, nozzle inclination was optimized by the different selected air incidence angles (*θ*) in simulation, and *θ* = π/4 was advised. Then, the grey relational analysis based on the orthogonal test method was used to analyze the grey relational grade for parameters and obtain the optimal parameter combination, i.e., at electrolyte velocity 5.5 m/s, gas velocity 160 m/s, and voltage 8 V. Finally, the optimization result was verified experimentally.

## 1. Introduction

Modern tribology [1,2], aerodynamics [3], and bionics [4] all confirm that metal surfaces with morphologies comprising microstructures of tens to hundreds of microns across can significantly improve equipment performance; for example, reducing friction, wear, and frictional resistance, improving stiffness, and via the removal of undesirable surface adhesion properties and seizures. Devaiah et al. [5] investigated the effect of a micropit on cutting performance of tools and found that it reduced the cutting force of tools and changed chip shape. Li et al. [6] filled the micropits with MnS_2_ powder with different incidence rates, and found that the fraction coefficient decreased from 0.18 to 0.1, and the wear decreased. Xiao et al. [7] mentioned that dimples in mechanical seals could enhance heat transfer by increasing the solid–fluid contact area and mixing, thus reducing the seal’s interface temperature by about 10%. Djamaï [8] found that thermo-hydrodynamic mechanical-face seals, which were equipped with a notched rotating face, were efficient to reduce friction. Zhuang et al. [9] studied microstructure heat exchangers with different forms of microstructures and different aspect ratios and found that the heat transfer performance improved with the increase of aspect ratio of microstructures.

Much attention has been paid to the manufacturing of textures on metals, and Electrochemical machining (ECM) is one of the nontraditional methods to machine materials into complex shapes with high precision. In ECM, the material was removed by electrolysis. Compared with other microstructure machining methods, ECM has advantages of no mechanical or thermal stress, no burrs or distortion of features [10], high surface quality, and high material removal rate (MRR), regardless of material hardness. It has many applications, one of which is electrochemical jet machining (EJM). EJM can control an electrolyte jet with a high velocity, concentrating on the local range for ejecting the electrolyte through a nozzle. Therefore, only the workpiece material exposed to the jet is removed by anodic dissolution. It is widely used in the machining of microstructures due to its high localization, large aspect ratio of machining morphology, and having no special requirements on tool shape. Liu et al. [11] investigated abrasive composite EJM by adding a solid micro-abrasive into the electrolyte. The anode dissolution and micro-abrasive erosion worked together to remove materials and improve the surface texture processing efficiency. Kunieda et al. [12] proposed an EJM application using a flat electrolyte jet, which offered a new idea for micromilling and electrochemical turning. Schubert et al. [13] applied superimposing multidimensional motions to a fabricated 3D complex structure with spiral geometry by EJM. Clare et al. [14] studied the influence of different parameters (jet angle, electrolyte, and current density) on the machining morphology by using a nozzle that could change the jet angle and produced complex structures with better precision. Hackert et al. [15] established a fluid dynamic model with COMSOL Multiphysics to simulate the formation and change of the liquid beam, and demonstrated the material removal process in EJM. Zhao et al. [16] established a multiphysics model to simulate EJM on inclined planes and studied the distribution of current density under different jet angles and workpiece configurations, and experimental verification was conducted to confirm the analysis. To reduce the undercutting, Chen et al. [17] proposed a method of conductive mask EJM for generating a micro-dimple. Speidel et al. [18] investigated different electrolytes for the purpose of enhancing the effect of processing. However, in EJM, severe stray corrosion around the machining area with opposite electrodes, which is caused by the hydraulic jump, would seriously affect the accuracy of microstructures. To solve this problem, Wang et al. [19,20] proposed a new processing method of air-shielding electrochemical micromachining (AS-EMM), which made a layer of compressed gas film coat the liquid beam coaxially and control the electrolyte to focus precisely on the workpiece area where the tool electrode was directly opposite.

Previous studies [19,20] have proved that AS-EMM can significantly reduce stray corrosion and improve the surface processing quality. However, the optimization of the nozzle inclination and machining parameters affecting the machining performance is essential. In this study, the nozzle was firstly optimized in simulation, based on the different incidence angles. Then, Grey relational analysis based on the orthogonal test method was used to develop a mathematical model for experimental results, and the built model was used as the objective functions for the multi-objective optimization. The process parameters were optimized to get the better performance of surface roughness and the micro pit’s aspect ratio. Finally, the optimization result was verified experimentally.

## 2. Schematic of the Air-Shielding Electrochemical Micromachining (AS-EMM)

**Figure 1:**
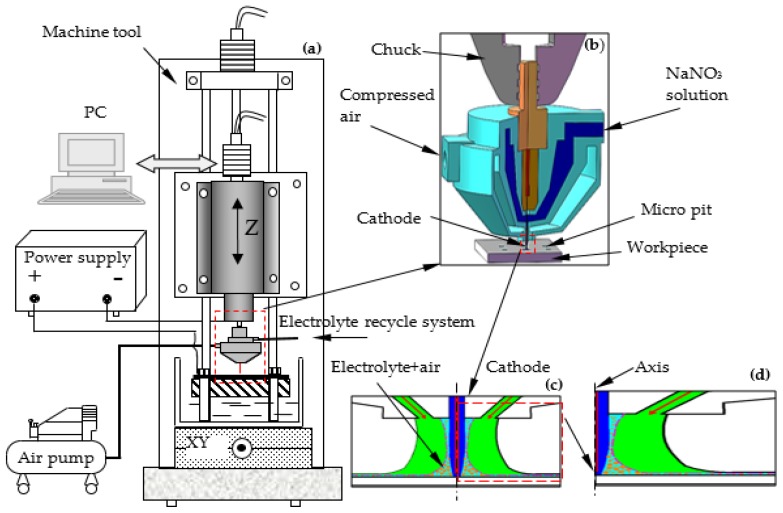
Schematic of (**a**) air-shielding electrochemical micromachining (AS-EMM) experimental set-up; (**b**) nozzle structure; (**c**) the flow field in interelectrode gap; (**d**) research zone.

The schematic of AS-EMM is shown in Figure 1. The experimental configuration includes the machining tool, the control system (computer), the electrolyte recycle system, an air pump, power supply, and a nozzle. The air pump and electrolyte recycle system provide gas and electrolyte in the interelectrode gap (IEG) with a certain amount of pressure, respectively. The machine tool is controlled by the control system. The cathode is fixed in the spindle of the machine tool through the nozzle. Material on the workpiece surface is eroded via the power supply when connected.

## 3. Optimization of Air Incidence Angle

### 3.1. Model Description

**Figure 2:**
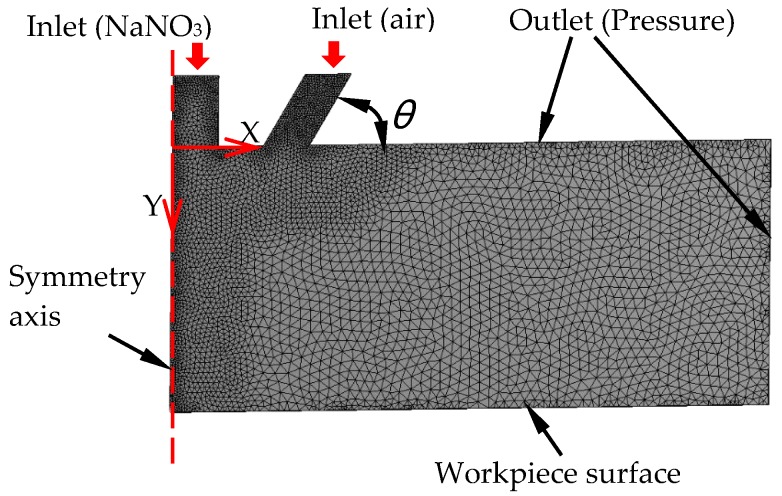
The structure model in the flow field.

In AS-EMM, nozzle inclination influences the flow field in the machining gap and then the machined micro pits. The optimization of the nozzle was done in simulation. The numerical model was created, and Fluent was employed to analyze the flow field characters. Due to the axis symmetry, the model was built as in Figure 2. There were 30,854 triangle units in total in this model. Considering the electrolyte and air in the machining gap, the mixture model was selected. The inlets’ boundary condition for air and the electrolyte were 0.15 MPa and 0.1 MPa, respectively. The initial outlet pressure was 0 MPa. The distance from the outlet of electrolyte to the workpiece surface is 5 mm. The effect of the compressed air incidence angle on air void fraction and velocity in the flow field was investigated. In AS-EMM, lower air fraction in the machining area can improve conductivity of flow, so that the MRR is improved. Higher velocity of the electrolyte is helpful for removing the products in machining and updating the electrolyte, which can improve machining localization and MRR. As a result, the specific air incidence angle leads to lower air void fraction, and a higher flow velocity is required in simulation.

### 3.2. Effects of Compressed Air Incidence Angle on Void Fraction (Air) in the Flow Field

**Figure 3:**
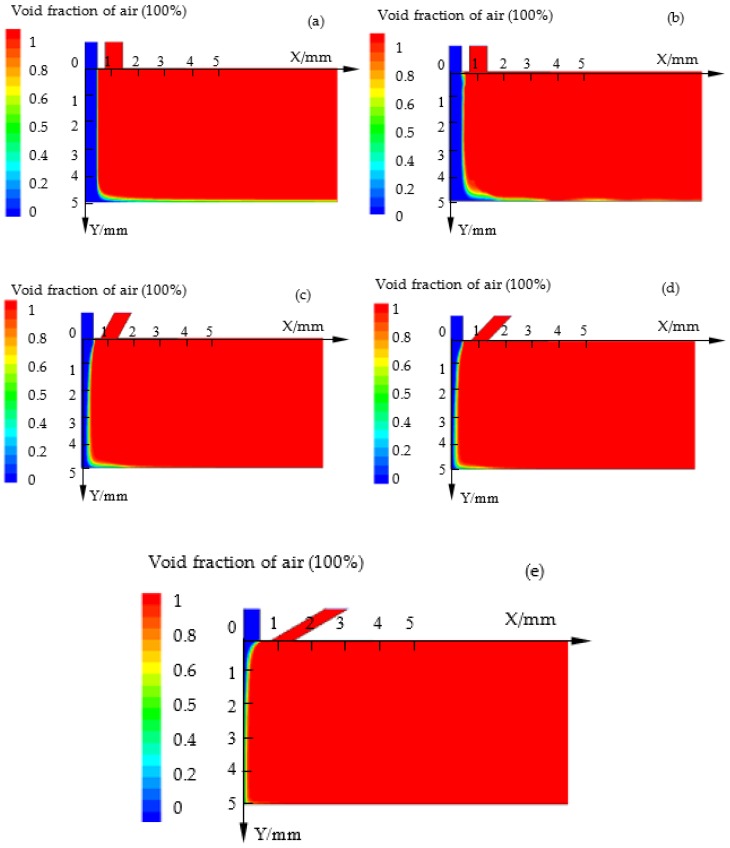
Contours of void fraction (air) in flow field by simulation at different incidence angles (*θ*): (**a**) No compressed air; (**b**) *θ* = π/2; (**c**) *θ* = π/3; (**d**) *θ* = π/4; (**e**) *θ* = π/6.

Figure 3 shows the contours of void fraction (air) in the flow field by simulation at different incidence angles (*θ*) of compressed air. Blue and red colors represent the electrolyte and air, respectively. The cloud charts indicate that the electrolyte beam flowing into the machining gap was confined and becomes thinner with the decrease of the incidence angle (*θ*). Even, under the condition of *θ* = π/6, the electrolyte beam disappears and becomes a mixture of electrolyte and air. That means that more air flows into the electrolyte beam and forms a mixed flow field to spray to the workpiece surface. In the machining process, more energy was expected to focus on the region where the material was removed. If there is more air mixing into the electrolyte, the void fraction of air would increase, which would lower the conductivity of the electrolyte, and so MRR decreases.

**Figure 4:**
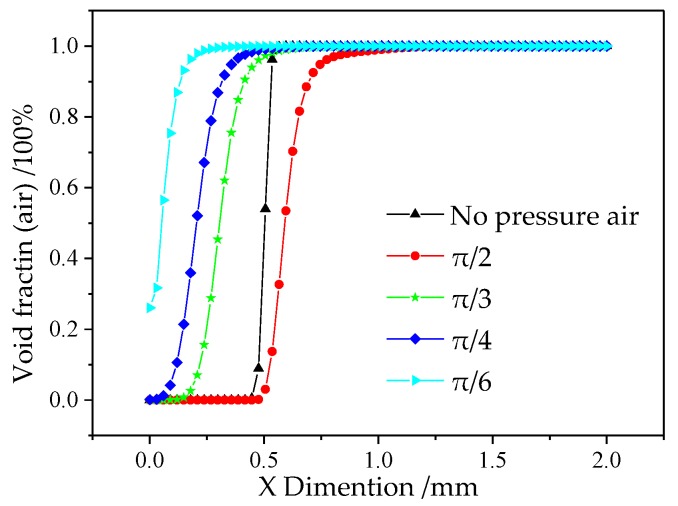
The extracted void fraction (air) (Y = 4, 0 < X < 2) at different incidence angles.

In order to obtain the value of the void fraction of air in the flow field, the data (Y = 4, 0 < X < 2) were extracted (see Figure 4) from the simulation model. The curve denotes that the void fraction of air increases, surrounding the symmetry Y axis, with the decrease of the incidence angle. When the incidence angle is larger than π/4, the focused effect is limited. However, under the condition of *θ* = π/6, there is no pure electrolyte at the bottom of the cathode electrode. The electrolyte is a mixture of NaNO_3_ and air, and this would lead to low MRR, which was not expected in the experiments. Therefore, based on the discussions above, the air incidence angle of *θ* = π/4 was advised.

### 3.3. Effects of Compressed Air Incidence Angle on Velocity in the Flow Field

**Figure 5:**
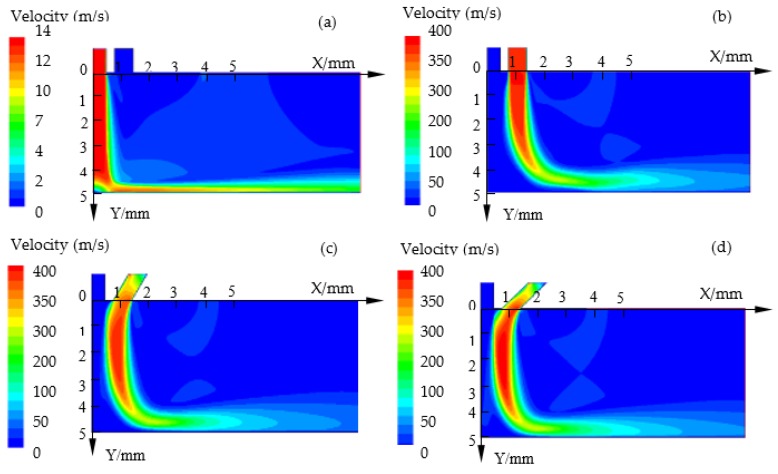

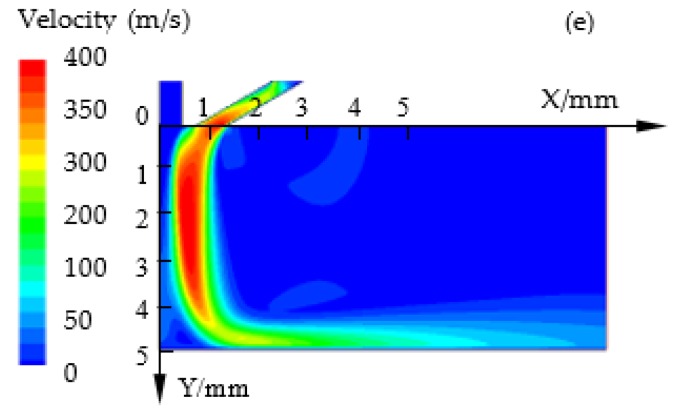
The electrolyte flow velocity in the interelectrode gap (IEG) in simulation at different incidence angles (*θ*): (**a**) No compressed air; (**b**) *θ* = π/2; (**c**) *θ* = π/3; (**d**) *θ* = π/4; (**e**) *θ* = π/6.

**Figure 6:**
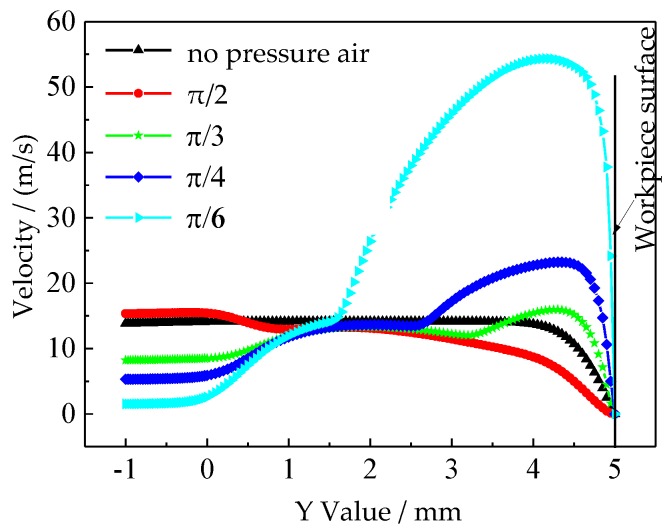
Graphs of velocity along the Y axis (X = 0) at different incidence angles (*θ*).

In EMM, the products (sludge, gas, and heat) in the machining gap should be removed at a certain electrolyte velocity. Timely renewal of the electrolyte can improve the machining speed and stability. It is also very important to remove the machining products and renew the electrolyte in the machining gap in AS-EMM. Figure 5 shows the contours of velocity in IEG by simulation at different incidence angles (*θ*). It can be seen that the NaNO_3_ solution was shielded more with the decrease of the incidence angle. The velocity along the Y axis varies when the applied compressed air is different. Figure 6 depicts the graphs of velocity along the Y axis (X = 0) at different incidence angles. It shows that the velocity clearly increases with the decrease of incidence angle (*θ*) and it can be twice the velocity without compressed air (see Figure 5d,e, *θ* = π/4 and π/6). The electrolyte would spray to the workpiece surface (Y = 5) at a higher speed and this would enhance the electrolyte renewal and discharge of the products. However, considering the void fraction of air, the smaller air incidence angle is a disadvantage to material removal, and *θ* = π/4 is more appropriate.

## 4. Experimental Design and Machining Results

The optimization of the processing parameters was carried out based on the advised nozzle inclination with the air incidence angle of π/4.

### 4.1. Design of Experiment

It was found that electrolyte velocity, gas velocity, and voltage had a great influence on machining results according to the preliminary experiments. In order to get the optimum processing parameter combination, an orthogonal test method was employed to design the experiments beforehand. The orthogonal test method is a kind of experimental design method to study multifactors and multilevels [21]. According to orthogonality, some representative points are selected from all experiments. It can greatly reduce the duration of the experiment and help get enough data rapidly. Therefore, a three-factor and four-level orthogonal experiment was design by Statistical Product and Service Solution (SPSS). The factors including electrolyte velocity, gas velocity and voltage, and their levels are shown in Table 1.

### 4.2. Experimental Details

The experimental condition is shown in Table 2. SS304 stainless steel (Mucheng, Foshan, China) and tungsten bar (Metalline, Luoyang, China) were selected as the workpiece material and tool material, respectively. Experiments were done to study the relationship between the machining parameters, including electrolyte velocity, gas velocity, and voltage, and the objectives, including the aspect ratio and the roughness (*Ra*) of the micro pit. Aspect ratio can be defined as *λ* = *H*/*D* (*H* is the depth, and *D* is the diameter of micro pit). The profiles of pit were measured by a 3D profilometer (Olympus LEXT4500, Olympus, Tokyo, Japan). The roughness was measured by a surface roughness meter (SJ-411, Mitutoyo, Kawasaki, Japan) and its uncertainty is less than 5%.

### 4.3. Experimental Results

**Figure 7:**
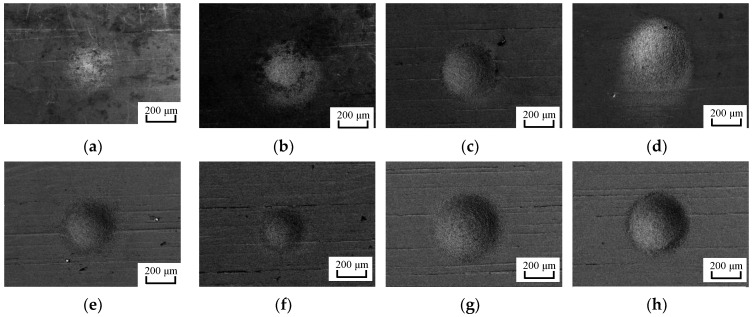

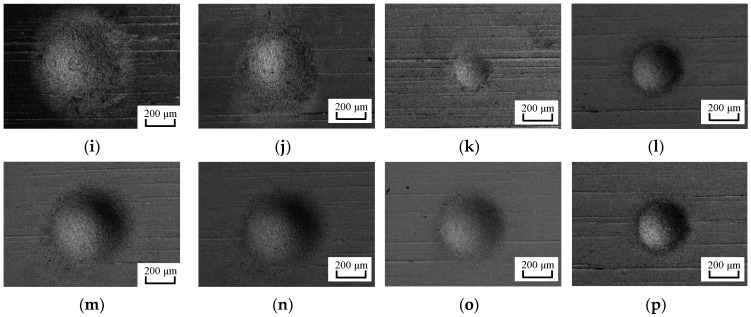
Morphology of machining results.

Scanning electron microscope (SEM) photos of the micro pits processed in AS-EMM, based on the conditions of Table 1 and Table 2, are shown in Figure 7a–p. The measured data of aspect ratio and roughness for the micro pits from Figure 7a–p are listed in Table 3 (No. 1–16). It can be seen that the micro pit size and surface morphology processed by AS-EMM vary greatly under different experimental parameters. The profile of the pit is not obvious when the voltage and electrolyte velocity are too small due to less material removal (Figure 7a). However, excessive voltage and electrolyte velocity lead to large pit size, serious stray corrosion, and reduced roughness, as shown in Figure 7i. The average detected aspect ratio and the roughness obtained with 5 points at different parameters are listed in Table 3.

## 5. Optimization of the Processing Parameters

The grey relational analysis is a method that can be applied to get the relationship between groups of data [22] and is fundamentally a simple and straightforward multicriteria decision-making technique [23]. To reduce errors caused by information asymmetry and better show influence of different parameters on machining results, grey relational analysis is introduced to determine the optimal parameters combination by analyzing the grey relational grade between parameters and responses.

### 5.1. Data Preprocessing

The grey relational analysis is based on the calculating the gray relational grade. It can be calculated according to the original processing results and then its relational coefficient can be computed. To simplify, it is necessary to make the original data dimensionless in units and orders of magnitude before calculating the grey relational grade.

The original sequence of 16 experimental results is set as $x_{i}(k)$, *i* = 1, 2……, 16; *k* = 1, 2.

Where *i* is the experimental sequence number, *k* = 1 represents the aspect ratio, and *k* = 2 represents the roughness.

As for the aspect ratio, in ECM, the larger the aspect ratio of pits is, the better localized the removal of the material is. Therefore, the pretreatment formula of aspect ratio is as follows:

(1) $$x_{i}^{*}(k)=\frac{x_{i}^{}(k)-\min x_{i}^{}(k)}{\max x_{i}^{}(k)-\min x_{i}^{}(k)}$$

For surface roughness, the lower the machined surface roughness value is, the better the machining quality is. Therefore, the pretreatment formula of surface roughness is as follows:

(2) $$x_{i}^{*}(k)=\frac{\max x_{i}^{}(k)-x_{i}^{}(k)}{\max x_{i}^{}(k)-\min x_{i}^{}(k)}$$

In Equations (1) and (2), $x_{i}(k)$,$x_{i}^{*}(k)$, $\max x_{i}(k)$, and $\min x_{i}(k)$ are the original sequence number, the sequence after data preprocessing, the maximum value, and minimum value in the original sequence, respectively. The dimensionless experimental results are shown in Table 4.

### 5.2. Correlation Coefficient

According to Table 4, the grey relational coefficients of aspect ratio and surface roughness are respectively obtained. The calculation formula can be described as

(3) $$\zeta (x_{0}(k),x_{i}^{*}(k))=\frac{\underset{i}{\min}\underset{k}{\min}|x_{0}(k)-x_{i}^{*}(k)|+\rho \underset{i}{\max}\underset{k}{\max}|x_{0}(k)-x_{i}^{*}(k)|}{\left|x_{0}(k)-x_{i}^{*}(k)|+\rho \underset{i}{\max}\underset{k}{\max}|x_{0}(k)-x_{i}^{*}(k)\right|}$$

where ζ is the grey relational coefficient, *ρ* is distinguishing coefficient, which is usually 0.5, and $\left|x_{0}(k)-x_{i}^{*}(k)\right|$ is the absolute difference between the expected sequence (expected value is 1) and the sequence after data processing, where $x_{0}(k)$ = 1 (*k* = 1,2), $\underset{i}{\min}\underset{k}{\min}|x_{0}(k)-x_{i}^{*}(k)|$ is the minimum value of the absolute difference sequence, and $\underset{i}{\max}\underset{k}{\max}|x_{0}(k)-x_{i}^{*}(k)|$ is the maximum value of the absolute difference sequence.

The grey relational grade can be obtained by

(4) $$\gamma_{i}=\frac{1}{N}\sum_{k=1}^{n}\zeta (x_{0}(k),x_{i}^{*}(k))$$

where $\gamma_{i}$ is the grey relational grade, and *N* is the number of performance characteristics.

The grey relational coefficient, grade of the aspect ratio, and roughness for each experiment were calculated by Equations (3) and (4) and listed in Table 5. Larger grey relational grade means a closer machining result to the desired value. From Table 5, the Exp. No. 12 has the highest grade 0.7887, which means the optimum parameter combination for the best structure morphology is A3B4C2.

**Figure 8:**
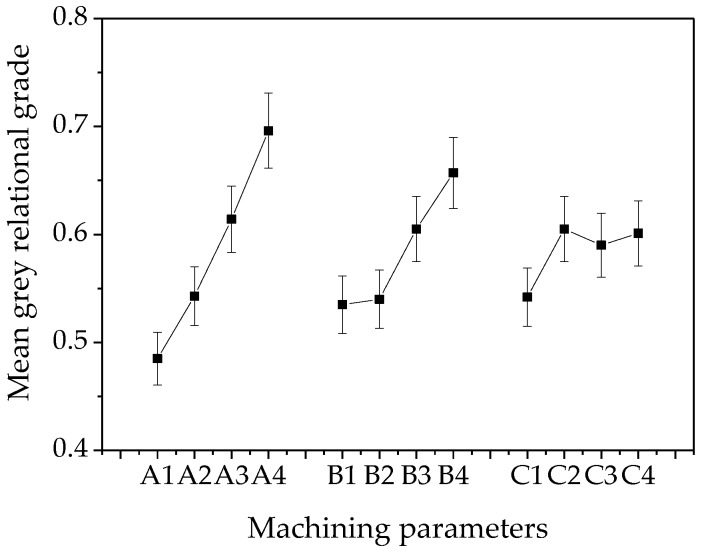
Mean grey relational grade of each parameter on machining results.

However, there are a total of 64 combinations in the three-factor and four-level experiments, among which there may be a parameter combination whose grey relational grade is higher than that of A3B4C2. Therefore, the influence of a single factor on the grey relational grade was calculated, which is shown in Figure 8. Based on Figure 8, the optimal combination of grey relational grade, which has the highest grade of each parameter, is A4B4C2, i.e., the electrolyte velocity is 5.5 m/s, the gas velocity is 160 m/s, and the voltage is 8 V.

### 5.3. Experimental Verification

**Figure 9:**
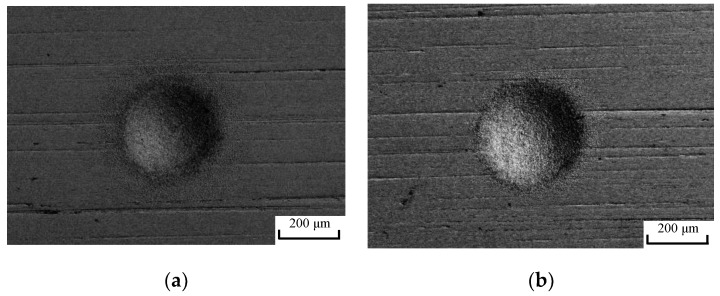
Micro pits machined using parameter combination of (**a**) group 12 and (**b**) the optimum group.

Verification experiments were carried out based on the optimum parameter combination of electrolyte velocity of 5.5 m/s, gas velocity of 160 m/s, and voltage of 8 V. The machining result is shown in Figure 9b. Figure 9 shows that the profile border of the machined pit by using the optimum parameters was sharper than that obtained by using the parameters in group 12. Its aspect ratio and roughness were 0.226 and 0.072, respectively, which is 3% higher and 11% lower than those of group 12. Verifying experiments denotes that the optimum parameters could be revised using the presented grey relational analysis method to realize a better machining morphology.

## 6. Conclusions

AS-EMM method was verified to be vital for machining microstructures on metal surface with high quality. In this paper, the nozzle inclination and machining parameters were optimized based on simulation and experiment analysis. Some conclusions are as follows:

1. Simulation results indicated that the void fraction (air) and velocity in the flow field increased with the decrease of nozzle inclination. According to the demonstrated model in this paper, the most appropriate nozzle inclination was *θ* = π/4;
2. The optimal parameter combination for the multi-objective was A4B4C2; i.e., at 5.5 m/s electrolyte velocity, 160 m/s gas velocity, and 8 V voltage, the micro pit with better performance in aspect ratio and roughness could be processed at these conditions;
3. The proposed method makes a contribution to the improvement of quality of the micro pits in AS-EMM. It is also effective for optimization of the structure design and machining parameters in other machining methods.

## Figures and Tables

**Table 1:** Parameters and their levels.

Factors	Symbol	Unit	Levels			
			1	2	3	4
Electrolyte velocity	A	m/s	4	4.5	5	5.5
Gas velocity	B	m/s	0	80	120	160
Voltage	C	V	6	8	10	12

**Table 2:** Parameters of experiment.

Parameters	Value
Machining gap	15 μm
Feed rate	60 μm·min^−1^
Electrode diameter	50 μm
Electrolyte concentration	10% NaNO_3_
Machining time	30 s
Duty ratio	50%
Frequency	100 kHz
Air incidence angle	π/4

**Table 3:** The detected machining results.

Exp. No	A	B	C	*λ*	Ra/μm	Exp. No	A	B	C	*λ*	Ra/μm
1	1	1	1	0.064	0.116	9	3	1	3	0.176	0.113
2	1	2	2	0.099	0.121	10	3	2	4	0.190	0.167
3	1	3	3	0.184	0.128	11	3	3	1	0.132	0.139
4	1	4	4	0.174	0.152	12	3	4	2	0.219	0.081
5	2	1	2	0.140	0.139	13	4	1	4	0.215	0.187
6	2	2	1	0.126	0.097	14	4	2	3	0.196	0.159
7	2	3	4	0.188	0.138	15	4	3	2	0.204	0.072
8	2	4	3	0.201	0.136	16	4	4	1	0.181	0.043

**Table 4:** Dimensionless experimental results.

Exp. No	Reference Sequence		Exp. No	Reference Sequence	
	*λ*	*Ra*		*λ*	*Ra*
1	0.000	0.493	9	0.724	0.514
2	0.225	0.458	10	0.811	0.139
3	0.772	0.410	11	0.438	0.333
4	0.711	0.243	12	1.000	0.736
5	0.491	0.333	13	0.977	0.000
6	0.397	0.625	14	0.853	0.194
7	0.797	0.340	15	0.901	0.799
8	0.886	0.354	16	0.752	1.000

**Table 5:** Grey relational coefficient and grey relational grade.

Exp. No	GRC		GRG	Rank	Exp. No	GRC		GRG	Rank
	λ	*Ra*				λ	*Ra*		
1	0.3333	0.4966	0.4149	16	9	0.6443	0.4286	0.5364	11
2	0.3920	0.4800	0.4360	15	10	0.7257	0.4513	0.5885	7
3	0.6864	0.4586	0.5725	8	11	0.4709	0.6145	0.5427	10
4	0.6340	0.3978	0.5159	12	12	1.0000	0.5774	0.7887	1
5	0.4954	0.4286	0.4620	14	13	0.9565	0.4977	0.7271	3
6	0.4534	0.5714	0.5124	13	14	0.7725	0.4777	0.6251	6
7	0.7115	0.4311	0.5713	9	15	0.8345	0.6341	0.7343	2
8	0.8145	0.4364	0.6254	5	16	0.6686	0.7294	0.6990	4
